# Associations of ACE Gene Insertion/Deletion Polymorphism, ACE Activity, and ACE mRNA Expression with Hypertension in a Chinese Population

**DOI:** 10.1371/journal.pone.0075870

**Published:** 2013-10-01

**Authors:** Qingfang He, Chunhong Fan, Min Yu, Gina Wallar, Zuo-Feng Zhang, Lixin Wang, Xinwei Zhang, Ruying Hu

**Affiliations:** 1 Zhejiang Provincial Center for Disease Prevention and Control, Hangzhou, China; 2 Zhejiang Medical College, Hangzhou, China; 3 Department of Epidemiology, Fielding School of Public Health, University of California Los Angeles, Los Angeles, California, United States of America; IPO, Inst Port Oncology, Portugal

## Abstract

**Background:**

The present study was designed to explore the association of angiotensin converting enzyme (ACE) gene insertion/deletion (I/D, rs4646994) polymorphism, plasma ACE activity, and circulating ACE mRNA expression with essential hypertension (EH) in a Chinese population. In addition, a new detection method for circulating ACE mRNA expression was explored.

**Methods:**

The research was approved by the ethics committee of Zhejiang Provincial Center for Disease Prevention and Control. Written informed consent was obtained prior to the investigation. 221 hypertensives (cases) and 221 normotensives (controls) were interviewed, subjected to a physical examination, and provided blood for biochemical and genetic tests. The ACE mRNA expression was analyzed by real time fluorescent quantitative Reverse Transcription PCR (FQ-RT-PCR). We performed logistic regression to assess associations of ACE I/D genotypes, ACE activity, and ACE mRNA expression levels with hypertension.

**Results:**

The results of the multivariate logistic regression analysis showed that the additive model (ID, DD versus II) of the ACE genotype revealed an association with hypertension with adjusted OR of 1.43(95% CI: 1.04-1.97), and ACE ID genotype with adjusted OR of 1.72(95% CI: 1.01-2.92), DD genotype with adjusted OR of 1.94(95% CI: 1.01-3.73), respectively. In addition, our data also indicate that plasma ACE activity (adjusted OR was 1.13(95% CI: 1.08-1.18)) was significantly related to hypertension. However, the plasma ACE mRNA expressions were not different between the cases and controls.

**Conclusion:**

ACE I/D polymorphism and ACE activity revealed significant influence on hypertension, while circulating ACE mRNA expression was not important factors associated with hypertension in this Chinese population. The detection of circulating ACE mRNA expression by FQ-RT-PCR might be a useful method for early screening and monitoring of EH.

## Introduction

Essential hypertension (EH) is a major public health burden worldwide and has been steadily increasing for the past several years in China. According to the Chinese National Nutrition and Health Survey in 2002, the prevalence of EH among adults (over 18 years old) was found to be 18.8% in China. EH is a multifactorial complex disorder caused both by genetic and environmental factors. Approximately 30% of the interindividual variability in blood pressure is estimated to be genetically determined. Numerous studies have been focused on the role of genetic variation in genes implicated in the renin-angiotensin system (RAS), particularly the angiotensin-converting enzyme (ACE) gene.

RAS is one of the most important systems in blood pressure regulation. ACE is the core enzyme in the RAS catalyzing the production of angiotensin II (Ang II). Ang II is the key effector in RAS which functions as a regulator of blood pressure, water-salt balance, and angiotasis. A 287 bp insertion/deletion (I/D, rs4646994) polymorphism in intron 16 of the ACE gene gives rise to the homozygotes II and DD, and the heterozygote ID. Some studies have shown an association between the DD genotype and hypertension [1-3], other studies, however, have failed to confirm this [4-6]. This I/D polymorphism is reported to account for 20-50% of the interindividual variation in ACE activity, and usually II, ID and DD genotype are associated with low, intermediate and high ACE levels, respectively [7-10]. But there are still 50–80% of the variation is due to other factors [11-13].

The association of the D-allele or DD genotype with significantly higher ACE mRNA expression in tissue has been clearly documented [14,15]. However, reports of the ACE gene mRNA expression in peripheral blood are rare. The objective of this study is to explore the association of the ACE gene insertion/deletion polymorphism, plasma ACE activity, and ACE mRNA expression in peripheral blood with essential hypertension in the Chinese population, and in addition, to apply the TaqMan real time fluorescent quantitative RT-PCR to the detection of the mRNA expression of ACE in peripheral blood.

## Subjects and Methods

### Ethics statement

The research was approved by the ethics committee of Zhejiang Provincial Center for Disease Prevention and Control. Written informed consent was obtained prior to the investigation.

### Subjects

Subjects came from a community baseline investigation on chronic disease in Zhejiang Province in recent years. Seven counties were selected in the investigation based on their mean income and typical socio-economic status in a multiple-stage stratified cluster randomization sampling method, then one community was selected from each above county in a simple randomization sampling rule. Several small residential areas were randomly sampled in each community and a cross-sectional study was carried out in these seven communities of the population aged 35 years and over.

A total of 9485 residents were investigated. Among them 1857 residents were confirmed EH according to the diagnostic criteria of EH established by World Health Organization (WHO) / International Society of Hypertension (ISH) in 1999. Cases were selected among the 1857 EH patients if they had been untreated for 2 weeks prior to the conduct of the study and if they had no diabetes, no history of stroke, no coronary heart disease, neither gout. There are totally 221 cases who met the above criteria. The controls were 1:1 matched with the cases by small residential area, gender and age (±5 years). 221 controls were selected in healthy individuals without EH, diabetes, stroke history, coronary heart disease, or gout. Written informed consent was obtained prior to the investigation.

### Instruments

The main instruments used including a fluorescence quantitation polymerase chain reactor (BioRad, Hercules, USA), an auto-biochemical analyzer (Beckman, Brea, USA), a biosafety cabinet (Thermo, Barrington, USA), and an ultraviolet Nano-drop1000 spectrophotometer (Thermo, Wilmington, USA).

### Field investigation

All the investigators for this study received training and passed qualifying examinations. The field investigation included administration of an epidemiological questionnaire, a physical examination, and laboratory tests.

The epidemiological questionnaire obtained general demographic characteristics, family history, medical history, and other factors including smoking, drinking, physical health, diet(food frequency questionnaires), and mental health.

Investigators performed physical examinations to obtain height, weight, waist circumference (WC), systolic blood pressure (SBP), and diastolic blood pressure (DBP) of participants. The body mass index (BMI) was calculated as follows: BMI= weight (kg) / height^2^ (m^2^)

Participants were asked about their smoking status. Those who responded as abstained smoking means they smoked but abstained for more than one year. Occasionally smoking was defined as those who occasionally smokes but does not meet the frequently smoking standard. Frequently smoking was defined as smoking every day for more than one year. People who did not smoke were defined as never smokers.

While people who did not drink were categorized as never drinking, abstained drinking was labeled for those who drank but abstained for more than one year. Occasionally drinking was defined as those who occasionally drinks but does not meet the frequently drinking standard. Frequently drinking was defined as consumption of alcohol every day for more than one year.

Individuals with mild occupational physical activities included those who work in an environment such as an office, retail or school. Those with moderate occupational physical activities included work in construction or its equivalent in physical activity. Individuals with heavy occupational physical activities included those that work as non-mechanized farming laborers, steel workers, dancers, or equivalent. Very heavy occupational physical activities were defined as those who work as foundry labors, lumbering workers. Since the number of very heavy occupational physical activities is only 1, it was combined with heavy occupational physical activities as one group.

Similarly, since the number of very high stress of work and life is also only 1, it was combined with high stress of work and life as one group.

A 5mL fasting EDTA-anticoagulated venous blood sample was collected from each subject. Plasma was subsequently separated by centrifugation and subjected to automated biochemical analysis to measure levels of total cholesterol (TC), triglycerides (TG), high-density lipoprotein cholesterol (HDLC), fasting blood glucose (FBG), total protein (TP), albumin (ALB), uric acid (UA), and plasma ACE activity. The remaining blood cells were stored at -80°C for the gene detection assay.

### ACE gene I/D polymorphism

QIAamp DNA Blood Mini kits (Qiagen, Düsseldorf, Germany) were used to extract DNA from blood cells. Primers used for the ACE I/D polymorphism analysis were as previously described [16]: Both forward and reverse primers were synthesized by Invitrogen; forward primer 5’-CTG GAG ACC ACT CCC ATC CTT TCT-3’; reverse primer 5’-GAT GTG GCC ATC ACA TTC GTC AGA T-3’. The PCR products were analyzed on 2% agarose gels with a 100-bp DNA Ladder (Promega), and images were acquired and analyzed using a gel imaging system.

The ACE gene I/D polymorphism transmission models were considered: (1) additive (II = 0, ID =1, and DD = 2), (2) dominant (DD + ID versus II), and (3) recessive (DD versus ID + II) models of the D allele.

### Expression of ACE mRNA

#### Real-time RT-PCR primer and probe design

Real-time fluorescent quantitative RT-PCR was used to measure ACE mRNA expression. The sequence encoding the ACE gene was retrieved from GenBank. The real-time fluorescent quantitative RT-PCR primers and TaqMan probes used to detect the human ACE gene and the internal reference gene (β-actin) were designed using the Primer Express software (PE Applied Biosystems).

#### ACE gene(NM_000789.3)

Forward primer: 5’-AGC CAA CCA CAC CCT GAA GT-3’,

Reverse primer: 5’-GTG TTC TGC AAC TGG TTC ACA TC-3’,

Fluorescent probe: 5’-CGG CAC CCA GGC CAG GAA GTT-3’, labled by dye FAM.

#### Internal reference gene (β-actin, NM_001101.3)

Forward primer: 5’-CCG TCT TCC CCT CCA TCG-3’,

Reverse primer: 5’-GTC CCA GTT GGT GAC GAT GC-3’,

Fluorescent probe: 5’-CCA GGG CGT GAT GGT GGG CA-3’, labled by dye FAM.

#### Real-time fluorescent quantitative RT-PCR analysis

Total RNA from blood cells collected from the cases and controls was extracted using QIAamp RNA Blood Mini kits (Qiagen, Düsseldorf, Germany). The total RNA concentration was determined using an ultraviolet spectrophotometer and 1% agarose gel electrophoresis. One-Step RT-PCR Kits (Perfect Real Time, TaKaRa) were used for this analysis. The reaction was run in an MJ Option 2 fluorescence quantitation polymerase chain reactor (BioRad, USA) in a volume of 25 µL. The following protocol was used: 40 cycles of 42°C for 30 minutes, 95°C for 2 minutes, 95°C for 5 seconds, and 54°C for 35 seconds. Reactions for each RNA sample were performed in duplicate under the same conditions. No template controls were used throughout the experiment.

#### Creation of the standard curve [17].

The total RNA (concentrated in cases of low RNA concentration) from one randomly selected subject was subjected to five-fold serial dilutions (1×, 5×, 25×, 125×, and 625×). The serial dilutions served as the reference standard and were subjected to real-time fluorescent quantitative RT-PCR along with the other samples. The copy numbers of the reference standard for the MJ Option 2 fluorescence quantitation polymerase chain reactor were defined proportionally on the basis of serial dilutions (1-, 5-, 25-, 125-, and 625-fold dilution) to 625, 125, 25, 5 and 1 in this experiment.

The C_T_ values of the serially diluted reference standards and the previously defined copy numbers were imported into Excel. The relative quantitative standard curves for the ACE and β-actin genes were set by using the base 10 logarithm of the copy number on the x-axis and the C_T_ value on the y-axis.

The copy numbers of expressed ACE and β-actin in each sample were obtained by separately inputting the averages of the two C_T_ values from each sample into the standard curves of the ACE and β-actin genes. The relative expression levels of the standardized samples were obtained by dividing the ACE gene copy numbers by the β-actin copy numbers. The standardized relative expression levels of the EH group and the control group were compared.

### Statistical analysis

All data were analyzed by using SPSS 16.0 software. The continuous variables were presented as the means ± SD. Comparisons of continuous variables were performed using *t*-tests and the means of multiple groups were compared with one-way ANOVA analysis. Relative ACE mRNA expression levels (non-normal distribution data) were log-transformed to normal distribution. The χ^2^ test was used to compare categorical variables. The univariate logistic regression analysis was performed to assess associations with known and suspected risk factors (multi-categorical variables transformed into dummy variables) between the cases and controls and group variable (cases=1, controls=0) was used as the dependent variable (sls=0.1, sle=0.05). All the significant factors from the univariate regression analysis and all the professionally known risk factors were controlled in the multivariate logistic regression analyses.

## Results

### The description of the age and gender for the cases and controls

As shown in Table 1, there were 221 cases and 221 controls, with 83 males and 138 females respectively. The mean age for cases was 58.2±11.6 and for controls was 58.2±11.6 (OR=1.00(95% CI: 0.99-1.02)). When age was divided into 35^~^45, 45^~^55, 55^~^65, and >65 four groups, there were 26, 64, 64, 67 individuals in each group both in cases and controls.

**Table 1:** The univariate logistic regression analysis of the risk factors between the cases and controls.

Variables	Cases(221)	Controls(221)	*OR*_c_(95% CI)
Gender(M/F)	83/138	83/138	1.00 (0.68-1.47)
Age (years) ± SD	58.2±11.6	58.1±11.4	1.00 (0.99-1.02)
Smoking			
never	172	156	1.00
abstained	10	7	1.30 (0.48-3.49)
occasionally	8	11	0.66 (0.26-1.68)
frequently	31	47	0.60 (0.36-1.00)
Drinking			
never	151	150	1.00
abstained	6	4	1.49 (0.41-5.39)
occasionally	37	32	1.15 (0.68-1.94)
frequently	27	35	0.77 (0.44-1.33)
Occupational physical activities			
mild	142	152	1.00
moderate	65	58	1.20 (0.79-1.83)
heavy/ very heavy	14	11	1.36 (0.60-3.10)
Types of cooking oil			
vegetable oil only	82	142	1.00
mainly vegetable oil	76	65	2.03 (1.32-3.11)
mainly animal oil	63	14	7.79 (4.11-14.78)
Daily consumption of vegetables			
below 1/2 kg	162	150	1.00
over 1/2 kg	59	71	0.77 (0.51-1.16)
Stress of work and life			
mild	96	104	1.00
moderate	110	98	1.22 (0.82-1.79)
high/ very high	15	19	0.86 (0.41-1.78)
BMI (kg/m^2^) ± SD_1_	24.04±3.23	22.16±3.02	1.22 (1.14-1.30)
18.5~24	103	146	1.00
<18.5	6	19	0.45 (0.15-1.16)
24~28	91	48	2.69 (1.75-4.14)
≥28	21	8	3.72 (1.59-8.73)
Waist Circumference (cm) ± SD_1_	82.2±9.6	76.4±8.8	1.07 (1.05-1.09)
normal(female<80,male<85)	104	154	1.00
abnormal(female≥80,male≥85)	117	67	2.59 (1.75-3.82)
Preference for salty foods			
no= 0	130	153	1.00
yes =1	91	68	1.58 (1.07-2.33)
Family history of hypertension			
no=0	134	156	1.00
yes=1	87	65	1.56 (1.06-2.31)
Family history of diabetes mellitus			
no=0	201	215	1.00
yes=1	20	6	3.57 (1.40-9.06)

_c_: Crude *OR*

_1_. Guidelines for Prevention and Control of Overweight and Obesity in Chinese Adults.

### Quantitative RT-PCR

Two clear bands of 28S and 18S at a brightness ratio of 2:1 (28S: 18S) in the agarose gel electrophoresis of total RNA suggested that the RNA was intact. The ratio of A260/A280 of the RNA was measured by the ultraviolet spectrophotometer as 1.9 to 2.1. The correlation coefficient *r* ^2^ of the standard curves of the ACE and β-actin genes were 0.9834 and 0.9994, respectively.

### Univariate Regression Analysis

A total of 32 independent variables (13 risk factors: smoking, drinking, occupational physical activities, types of cooking oil, daily consumption of vegetables, stress of work and life, BMI, BMI groups, WC, WC groups, preference for salty foods, family history of EH, family history of diabetes mellitus; 19 detected markers: ACE (rs4646994) genotype(additive model, dominant model, and recessive model), relative ACE mRNA, relative ACE mRNA groups, ACE activity, ACE activity groups, TC, TC groups, TG, TG groups, HDLC, HDLC groups, FBG, FBG groups, TP, TP groups, ALB, ALB groups, UA, UA groups) were analyzed using univariate regression analysis. Among the 13 risk factors, types of cooking oil, BMI, BMI groups, WC, WC groups, preference for salty foods, family history of hypertension and family history of diabetes mellitus revealed associations with hypertension. While among testing markers, relative ACE mRNA, relative ACE mRNA groups, ACE activity, ACE activity groups, TC, TC groups, TG, TG groups, FBG, and FBG groups showed significant difference between the cases and controls(Table 1, Table 2).

**Table 2:** The univariate logistic regression analysis of the distribution of the genotypes, relative mRNA expression levels of the ACE gene and clinical and biochemical test characteristics between the cases and controls.

Variables	Cases(221)	Controls(221)	*OR*_c_(95% CI)
ACE(rs4646994) genotype			
II	73	87	1.00
ID	97	95	1.22 (0.80-1.85)
DD	51	39	1.56 (0.93-2.62)
dominant model			
II	73	87	1.00
ID+DD	148	134	1.32 (0.89-1.94)
recessive model			
II+ID	170	182	1.00
DD	51	39	1.40 (0.88-2.23)
additive model			1.24 (0.96-1.61)
Relative ACE mRNA (log transformed)	1.55±0.72	1.38±0.72	1.41 (1.08-1.84)
low	51	73	1.00
median	70	74	1.35 (0.83-2.20)
high	100	74	1.93 (1.21-3.09)
ACE activity (U/L) ± *SD*	37±6	34±5	1.13 (1.09-1.18)
low	27	64	1.00
median	68	90	1.79 (1.03-3.10)
high	126	67	4.46 (2.60-7.64)
TC(mmol/L) ± SD_1_	4.92±1.02	4.62±0.88	1.40 (1.14-1.72)
normal(<5.18)	136	169	1.00
abnormal(≥5.18)	85	52	2.03 (1.35-3.07)
TG (mmol/L) ± SD_1_	1.88±1.41	1.45±0.98	1.48 (1.19-1.85)
normal(<1.70)	133	161	1.00
abnormal(≥1.70)	88	60	1.78 (1.19-2.65)
HDLC (mmol/L) ± SD_1_	1.25±0.34	1.23±0.28	1.16 (0.64-2.11)
normal(1.04~1.55)	108	131	1.00
abnormal(<1.04)	71	57	1.51 (0.98-2.33)
abnormal(≥1.55)	42	33	1.54 (0.92-2.60)
FBG (mmol/L) ± SD_2_	5.09±0.69	4.92±0.63	1.50 (1.12-2.01)
normal(<6.1)	200	211	1.00
abnormal(≥6.1)	21	10	2.22 (1.02-4.82)
TP (g/L) ± SD_3_	76.1±7.3	74.9±6.6	1.03 (1.00-1.05)
normal(64~83)	185	194	1.00
abnormal(<64)	9	6	1.57 (0.55-4.51)
abnormal(≥83)	27	21	1.35 (0.74-2.47)
ALB (g/L) ± SD_3_	42.6±4.0	42.0±3.6	1.04 (0.99-1.10)
normal(34~48)	202	205	1.00
abnormal(<34 or ≥48)	19	16	1.21 (0.60-2.41)
UA (µmol/L) ± SD_3_	280±80	276±74	1.00(1.00-1.00)
normal(female:155~357,Male: 208~428)	194	203	1.00
abnormal(Female: <155, Male: <208)	9	6	1.57 (0.55-4.49)
abnormal(Female: ≥357,Male: ≥428)	18	12	1.57 (0.74-3.34)

_c_: Crude *OR*

_1_. Chinese guidelines on prevention and treatment of dyslipidemia in adults.

_2_. Chinese guideline for type 2 diabetes mellitus.

_3_. National Guide to Clinical Laboratory Procedures(Third Edition)

### One-way ANOVA Analysis

Our one-way ANOVA analysis showed that both in cases and controls, subjects who carry the homozygotes II had the highest ACE activity, followed by homozygotes DD, and people carrying heterozygote ID had the lowest ACE activity. ACE activities differed significantly between II genotype and ID genotype both in cases (P<0.01) and in controls (P<0.01). Moreover, ACE activities between II genotype and DD genotype in cases differed significantly (P<0.05). For ACE mRNA expression, there were no significant differences among the II, ID and DD genotype groups(Table 3).

**Table 3:** Effect of ACE genotype on ACE activity between the cases and controls.

ACE genotype	Cases(221)				Controls(221)		
	II (n=73)	ID (n=97)	DD (n=51)		II (n=87)	ID (n=95)	DD (n=39)
ACE activity (U/L) ± *SD*	39±6	36±5^a^	37±6^b^		35±5	33±7^c^	34±5
Relative ACE mRNA expression(log transformed)	1.61±0.65	1.54±0.69	1.50±0.87		1.33±0.67	1.40±0.80	1.44±0.62

a P<0.01 between II genotype and ID genotype in cases;

b P<0.05 between II genotype and DD genotype in cases;

c P<0.01 between II genotype and ID genotype in controls;

### Multivariate Logistic Regression Analysis

After adjusted for age(continues), gender(male=1, female=2), BMI(continues), smoking(never=1, abstained=2, occasionally=3, frequently=4), drinking(never=1, abstained=2, occasionally=3, frequently=4), Waist Circumference(continues), preference for salty foods(no= 0, yes =1), family history of EH(no=0, yes=1), family history of diabetes mellitus (no=0, yes=1), types of cooking oil(vegetable oil only=1, mainly vegetable oil=2, mainly animal oil=3), CHOL(continues), TG(continues), FBG(continues), relative ACE mRNA (log transformed), and ACE activity(continues, for ACE(rs4646994) genotype) or ACE(rs4646994) genotype (for ACE activity(continues)), which were the significant factors from the univariate regression analysis and some of the professionally known risk factors, we found that the additive model (ID, DD versus II) of the ACE (rs4646994) genotype revealed a significant association with hypertension with adjusted OR of 1.43(95% CI: 1.04-1.97), and ID genotype with adjusted OR of 1.72(95% CI: 1.01-2.92), DD genotype with adjusted OR of 1.94(95% CI: 1.01-3.73), respectively. In addition, ACE activity was both significantly associated with ACE I/D polymorphism and hypertension (Table 4).

**Table 4:** The multivariate logistic regression analysis of the markers and risk factors between the cases and controls.

Variables	Cases(221)	Controls(221)	*OR*_a_(95% CI)
ACE(rs4646994) genotype			
Additive model			1.43 (1.04-1.97)
II	73	87	1.00
ID	97	95	1.72 (1.01-2.92)
DD	51	39	1.94 (1.01-3.73)
ACE activity (U/L) ± *SD*	37±6	34±5	1.13 (1.08-1.18)

_a_: Adjust for age(continues), gender(male=1, female=2), BMI(continues), smoking(never=1, abstained=2, occasionally=3, frequently=4), drinking(never=1, abstained=2, occasionally=3, frequently=4), Waist Circumference(continues), preference for salty foods(no= 0, yes =1), family history of EH(no=0, yes=1 , family history of diabetes mellitus (no=0, yes=1 , types of cooking oil(vegetable oil only=1, mainly vegetable oil=2, mainly animal oil=3), CHOL(continues), TG(continues), FBG(continues), relative ACE mRNA (log transformed), and ACE activity(continues, for ACE(rs4646994) genotype) or ACE(rs4646994) genotype (for ACE activity(continues)), which were the significant factors from the univariate regression analysis and some of the professionally known risk factors.

## Discussion

In this study, the distribution of the genotypes of the ACE gene was in Hardy-Weinberg equilibrium, indicating that the selected samples are representative. The potential role of the I/D polymorphism and thus of differential ACE plasma activity and ACE mRNA expression in the development of hypertension is still a matter of controversy [1-15,18,19]. We presented in this study that the ACE D allele was significantly associated with hypertensionin this Chinese population. The additive model (ID, DD versus II) of the ACE (rs4646994) genotype revealed an association with hypertension with adjusted OR of 1.43(95% CI: 1.04-1.97), and ID genotype with adjusted OR of 1.72(95% CI: 1.01-2.92), DD genotype with adjusted OR of 1.94(95% CI: 1.01-3.73), respectively (Table 4). Also the recessive model (DD versus II+ID) showed an association with hypertension with an adjusted odds ratio of II+ID 1.79(95% CI: 1.09-2.92) (data not shown in Table 4), indicating that the ACE gene I/D polymorphism appeared strong evidence to support the role of the D-allele and/or DD genotype as a risk factor for hypertension. These findings are in agreement with those of Ahmad Ali [2], who noted that hypertensive cases showed a significantly higher frequency of the ACE mutant D allele carriage than I allele carriage. Also, Zarouk et al [1] reported that DD genotype and the D allele are significantly associated with hypertension in Egyptian patients, A significant association between the I/D variant and hypertension was also found among Han Chinese through recent meta-analysis [3]. However, Kabadou [4] revealed that the ACE I/D polymorphism is not significant factor for hypertension in the Tunisian population. The study of Rasyid et al [5] did not support that the ACE I/D polymorphism associated with hypertension in a South Sulawesi Indonesian population. This inconsistency may be due to differences between studies in terms of ethnicity, gender, or publication bias (positive results being easier to publish).

It has been suggested that the increase in serum ACE activity in essential hypertension is not of pathophysiological or clinical significance [20]. Persu et al confirmed the hypothesis that membrane-bound ACE, instead of circulating ACE, was responsible for Angiotensin II generation and its cardiovascular consequences [21]. However, several reports suggested that the plasma ACE activity might be higher in adults with several cardiovascular disorders such as myocardial infarction, diabetic nephropathy, and carotid artery thickening [22-24]. In children, this critical enzyme has been found to be positively associated with blood pressure levels and contribute to the development of hypertension [25]. The data obtained in our report supports to the hypothesis that ACE activity and hypertension are associated. After multivariate regression analysis the plasma ACE activity revealed statistically significant difference between cases and controls with adjusted OR=1.13, 95%CI= 1.08-1.18(Table 4). Our analysis showed that both in cases and controls, ACE activities were highest in II carriers, then in DD carriers, and lowest in ID carriers. This result was consistent with part of Ljungberg’s [26] report that several individuals within the II group had considerably higher plasma ACE level than most DD carriers, but not in concordance with the great majority of the researches for most of them reported that ACE activities were significantly higher in patients with DD genotype versus the two other groups ID and II, and ID genotype usually showed intermediate levels and II genotype lowest levels [7-10]. Though ACE activities appeared to be affected by the I/D polymorphism, the I/D polymorphism was reported to be accounted for 47% of the total phenotypic variance of ACE activity, indicating that the ACE gene locus might be the major locus that determined ACE activity. However, despite the ACE I/D polymorphism, some other genetic variants, either inside or outside the ACE coding region, have been believed to influence levels of circulating ACE [11-13]. The contradiction emphasises the need for large sample sized studies.

The expression levels of ACE mRNA may be influenced by multiple factors, including developmental phases, growth conditions, lesion locations, and duration and conditions of storage. Therefore, mRNA expression levels of ACE among different tissues are incomparable. We thus explored the correlation of the EH and ACE gene I/D polymorphism with mRNA expression levels of the ACE gene with fresh blood samples. The univariate regression analysis of our study showed an association between hypertension and mRNA expression level of ACE (crude OR=1.41, 95% CI: 1.08-1.84), especially in the high mRNA expression level group (crude OR=1.93, 95% CI: 1.21-3.09), indicating a potential relationship between the aberrant mRNA expression of ACE and hypertension (Table 2). There are few reports on ACE mRNA expression level in blood and hypertension and less on the association of ACE mRNA expression level with hypertension in tissue [14]. Suehiro et al [15] reported that the D allele leaded to higher expression of the ACE mRNA and thus balanced the plasma ACE level. However, the evidence of the potential role of the I/D polymorphism to diferential ACE expression for the development of human hypertension was weak. In our study, the relative ACE mRNA expression showed no association with hypertension after adjusted in the multivariate regression analysis, and also revealed no significant difference among II, ID, DD genotypes in the cases and controls. The lack of effect of ACE mRNA expression on blood pressure might be attributable to homeostatic compensations [27,28]. Gene targeting experiments in mice provided additional important evidence on the importance of compensatory mechanisms to maintain normal blood pressures. The endothelial ACE expression was knocked out and plasma ACE activity was either fully or partially replaced by ACE expression targeted to the liver [29,30].

In a conclusion, EH involves interactions between genetic and environmental factors. previous studies illustrated that different populations with varying genes, lifestyles, dietary habits, environmental exposures,as well as the potential gene-environment interactions all contribute to the expression of hypertension [3,6,19,31]. The univariate regression analysis of our study revealed that types of cooking oil, BMI, WC, preference for salty foods, family history of hypertension, family history of diabetes mellitus, TG, CHOL, FBG, relative ACE mRNA, and ACE activity revealed associations with hypertension. After controlling these significant factors from the univariate regression analysis and some of the professionally known risk factors such as age, gender, smoking and dringking, finally plasma ACE activity and ACE I/D polymorphism revealed significant influence on hypertension, while circulating ACE mRNA expression was not an important factor affecting hypertension in the Chinese population. However, the small sample size (442) as well as limited information on potential risk factors may restrict the ability to make a detailed confounding adjustment for all factors essential for the study of hypertension. Therefore, the relationship between the ACE I/D polymorphism, plasma ACE activity, and ACE mRNA expression with EH is still inconclusive and must await larger scaled and further studies.

There are mainly two ways to quantitate gene expression levels: absolute quantification and relative quantification. Absolute quantification is an approach calculating the copy number of the initial template with the standard curves. Despite its accuracy and reliability, the reference standard is required to create the standard curve by cloning gene fragments and constructing the in vitro transcription system, which are complicated and difficult [32]. The classical 2^-△△CT^ relative quantification is a simple method of real-time quantitative PCR experiments for the analysis of gene expression profiles [33]. However, the calculated value is generally higher than the actual value and a greater error is expected. The gene expression levels can be detected and analyzed in a simple and accurate manner by adopting a novel and convenient analytical tool: the real time fluorescent relative quantitative RT-PCR introduced by Chiyu Zhang and colleagues [17]. In addition, the analysis of gene expression by use of Real Time RT-PCR requires a house keeping gene as the reference gene. In the process, all the samples are normalized and the expression levels of the target gene in different samples are subsequently compared. The stably expressed β-actin gene was chosen as the internal standard in our study. The correlation coefficient *r* ^2^ of the standard curves of the β-actin genes was 0.9994, and it thus was proved to be an ideal internal standard.

The quantitative detection of the mRNA expression of ACE is relatively accurate and can be reflective of the aberrant mRNA expression of ACE. Admittedly, a great deal of research on the validation and delimitation of the corresponding threshold is still lacking for the prediction of EH through the quantitative detection of the mRNA expression levels of ACE in peripheral blood. In spite of that, owing to the wide availability of peripheral blood samples and the high sensitivity, convenience, high-accuracy, and good reproducibility of the fluorescent quantitative RT-PCR method, the detection of the mRNA expression of ACE in peripheral blood by fluorescent quantitative RT-PCR can be of great significance to early screening, prevention, treatment, surveillance, and diagnosis of EH, and thus it deserves further investigation.
