# Correction: Associations of ACE Gene Insertion/Deletion Polymorphism, ACE Activity, and ACE mRNA Expression with Hypertension in a Chinese Population

**DOI:** 10.1371/journal.pone.0156564

**Published:** 2016-05-24

**Authors:** Qingfang He, Chunhong Fan, Min Yu, Gina Wallar, Zuo-Feng Zhang, Lixin Wang, Xinwei Zhang, Ruying Hu

The term “plasma” appears incorrectly throughout the article. The correct term should be “serum”.

There are additional errors in the text. The final paragraph in the “Field investigation” subsection of the Subjects and Methods section is incorrect. The correct text is: A 5mL fasting venous whole blood sample and a 3mL EDTA-anticoagulated blood sample were collected from each subject. Serum was subsequently separated by centrifugation from whole blood samples and subjected to automated biochemical analysis to measure levels of total cholesterol (TC), triglycerides (TG), high-density lipoprotein cholesterol (HDLC), fasting blood glucose (FBG), total protein (TP), albumin (ALB), uric acid (UA), and ACE activity. The EDTA-anticoagulated blood cells were for the gene detection assay.

The fifth sentence under the “Real-time fluorescent quantitative RT-PCR analysis” heading in the “Expression of ACE mRNA” subsection of the Subjects and Methods section is incorrect. The correct sentence is: The following protocol was used: 42°C for 30 minutes, 95°C for 2 minutes, then 40 cycles of 95°C for 5 seconds, and 54°C for 35 seconds.

In Table 3, the value for ACE activity (U/L) ± *SD* in the Controls ID column is incorrect. Please see the corrected Table 3 here.

**Table 3:** Effect of ACE genotype on ACE activity between the cases and controls.

ACE genotype	Cases(221)			Controls(221)		
	II (n = 73)	ID (n = 97)	DD (n = 51)	II (n = 87)	ID (n = 95)	DD (n = 39)
ACE activity (U/L) ± *SD*	39±6	36±5^a^	37±6^b^	35±5	33±4^c^	34±5
Relative ACE mRNA expression (log transformed)	1.61±0.65	1.54±0.69	1.50±0.87	1.33±0.67	1.40±0.80	1.44±0.62

^a^: P<0.01 between II genotype and ID genotype in cases;

^b^: P<0.05 between II genotype and DD genotype in cases;

^c^: P<0.01 between II genotype and ID genotype in controls;
